# Rare disease curative care expenditure-financing scheme-health provider–beneficiary group analysis: an empirical study in Sichuan Province, China

**DOI:** 10.1186/s13023-022-02524-1

**Published:** 2022-10-08

**Authors:** Jia Li, Lian Yang, Yitong Zhang, Hailun Liao, Yuan Ma, Qun Sun

**Affiliations:** 1 HEOA Group, School of Management, Chengdu University of Traditional Chinese Medicine, Chengdu, Sichuan Province China; 2 HEOA Group, School of Public Health, Chengdu University of Traditional Chinese Medicine, Chengdu, Sichuan Province China; 3 HEOA Group, Department of Medical Record Management, West China Second University Hospital, Sichuan University, Chengdu, Sichuan Province China

**Keywords:** Rare diseases, Curative care expenditure, SHA2011, Medical security system

## Abstract

**Background:**

Rare diseases impose a heavy economic burden on patients’ families and society worldwide. This study used the samples from Sichuan Province in China to estimate the curative care expenditure (CCE) of ten rare diseases, for supporting the prioritization of rare disease health policies.

**Methods:**

Multi-stage cluster sampling method was adopted to investigate 9714 rare disease patients from 1556 medical institutions in Sichuan Province. Based on the System of Health Accounts 2011, this study estimated the total CCE of 10 rare diseases, financing schemes, and their allocation among different medical institutions and groups of people.

**Results:**

In 2018, the total CCE of the ten rare diseases was $19.00 million, the three costliest rare diseases were Hemophilia ($4.38 million), Young-onset Parkinson’s disease ($2.96 million), and Systemic Sclerosis ($2.45 million). Household out-of-pocket expenditure (86.00% for outpatients, 41.60% for inpatients) and social health insurance (7.85% for outpatients; 39.58% for inpatients) were the main sources of financing CCE. The out-of-pocket expenditures for patients with Young-onset Parkinson’s disease, Congenital Scoliosis, and Autoimmune Encephalitis accounted for more than 60% of the total CCE. More than 80% of the rare disease CCE was incurred in general hospitals. The 40–59 age group accounted for the highest CCE (38.70%) while men spent slightly more (55.37%) than women (44.64%).

**Conclusions:**

As rare disease treatment is costly and household out-of-pocket expenditure is high, we suggest taking steps to include rare disease drugs in the National Reimbursement Drug List and scientifically re-design insurance coverage. It is also necessary to explore a multi-tiered healthcare security system to pay for the CCE of rare diseases and reduce the economic burden on patients.

**Supplementary Information:**

The online version contains supplementary material available at 10.1186/s13023-022-02524-1.

## Introduction

Rare diseases refer to diseases that have a low prevalence rate and are extremely uncommon. The definition of rare disease differs among countries, and there is no explicit official definition for rare disease in China [1–3]. In 2018, China issued *The Catalog of the First Group of Rare Diseases,* which contains 121 rare diseases, such as systemic sclerosis (SSc) and Young-onset Parkinson’s disease (YOPD) [4].

There are currently 6000–8000 internationally acknowledged rare diseases, which account for about 10% of human diseases and affect approximately 475 million people globally [5]. Eighty percent of rare diseases are genetic, and 50% of them start in childhood. However, treatment methods are available for only 5% of them [6]. Rare diseases are serious and difficult to diagnose and treat, which can easily lead to damaged tissues and organs, resulting in disability, mental disorders, deteriorated quality of life, and even death. Rare diseases put an extremely heavy burden on patients’ families and society and pose immense challenges in building and improving global public healthcare systems [7–9]. Research has shown that the annual curative expenditure for Hemophilia in the United States is $80,811–$632,088. European patients with Multiple Sclerosis (MS) spend an average of $47,888 annually on curative care [10]; in Australia, the expenditure on rare diseases accounts for 4.60–10.50% of the total curative spending, equivalent to the curative expenditure on diabetes or asthma in 2010 [11]. Some studies have examined the economic burden of rare diseases in China. In 2016, hospitalization expenditure on rare diseases was 4.30% of all hospitalization expenditure in Hong Kong [12]. Researchers in Taiwan have found that the health expenditures for patients with rare diseases grew sharply from $18.65 million in 2003 to $137 million in 2014 [13]. One study estimated the direct medical costs for 23 rare diseases in Shanghai and showed that the mean direct medical costs were about $1521 for inpatients and $168 for outpatients [14].

With a population of 1.411 billion, China has its share of rare diseases. It is estimated that there are at least 20 million people with rare diseases in China, the largest population of rare disease patients globally [15]. This large number of patients will inevitably result in an increased economic burden and social problems [16]. Many studies have investigated rare diseases in China; to the best of our knowledge, most of them have focused on prevention and clinical research while few have examined curative care expenditure and medical insurance payment for rare diseases [17]. The Health China Plan states that promoting the development of rare diseases in China is important for formulating a Healthy China Program. Hence, it is essential to establish an expenditure calculation framework that can help estimate curative expenditure accurately, clarify who pays for the curative expenditure, and what the curative expenditure is spent on.

The System of Health Accounts 2011 is the global framework for national health accounts and has been widely adopted by the European Union, member states of the Economic Cooperation Organization, and other countries [18]. A study of CCE in 195 countries showed that global health financing had increased steadily between 1995 and 2016 and was expected to continue increasing in the future [19]. Indian scholars used the SHA2011 framework for preparing health accounts for Punjab state, which showed the CCE in Punjab was INR 134,680 million (US$ 2245 million) which was 4.02% of its gross state domestic product (GSDP) in 2013–14 [20]. The Chinese National Health Commission adopted SHA2011 in 2010. After mapping the contents of the system to the Chinese context, the China Health Cost Accounting Team conducted accounting at monitoring sites across the country based on SHA2011, to confirm the feasibility and scientific validity of the methodology in China. Our study was based on this work [21]. We used samples from Sichuan Province, which has a medium level of economic and medical development, to calculate the curative care expenditure (CCE) of the top 10 costly rare diseases and analyze the financing sources, distribution among healthcare providers, and allocation among different populations. Our study findings are expected to provide references for policymakers concerning the effective control of rare diseases and rational allocation of medical resources to reduce the economic burden on patients and protect their rights.

## Methods

### Data sources

The statistical data were obtained from the Sichuan Province Statistical Yearbook (2018), Sichuan Health Financial Statistical Yearbook (2018), and Sichuan Health Statistical Yearbook (2018). The data on patient’s medical expenditure were collected from the Hospital Information System (HIS) of 1556 medical institutions in Sichuan Province.

### Study sample

**Fig. 1:**
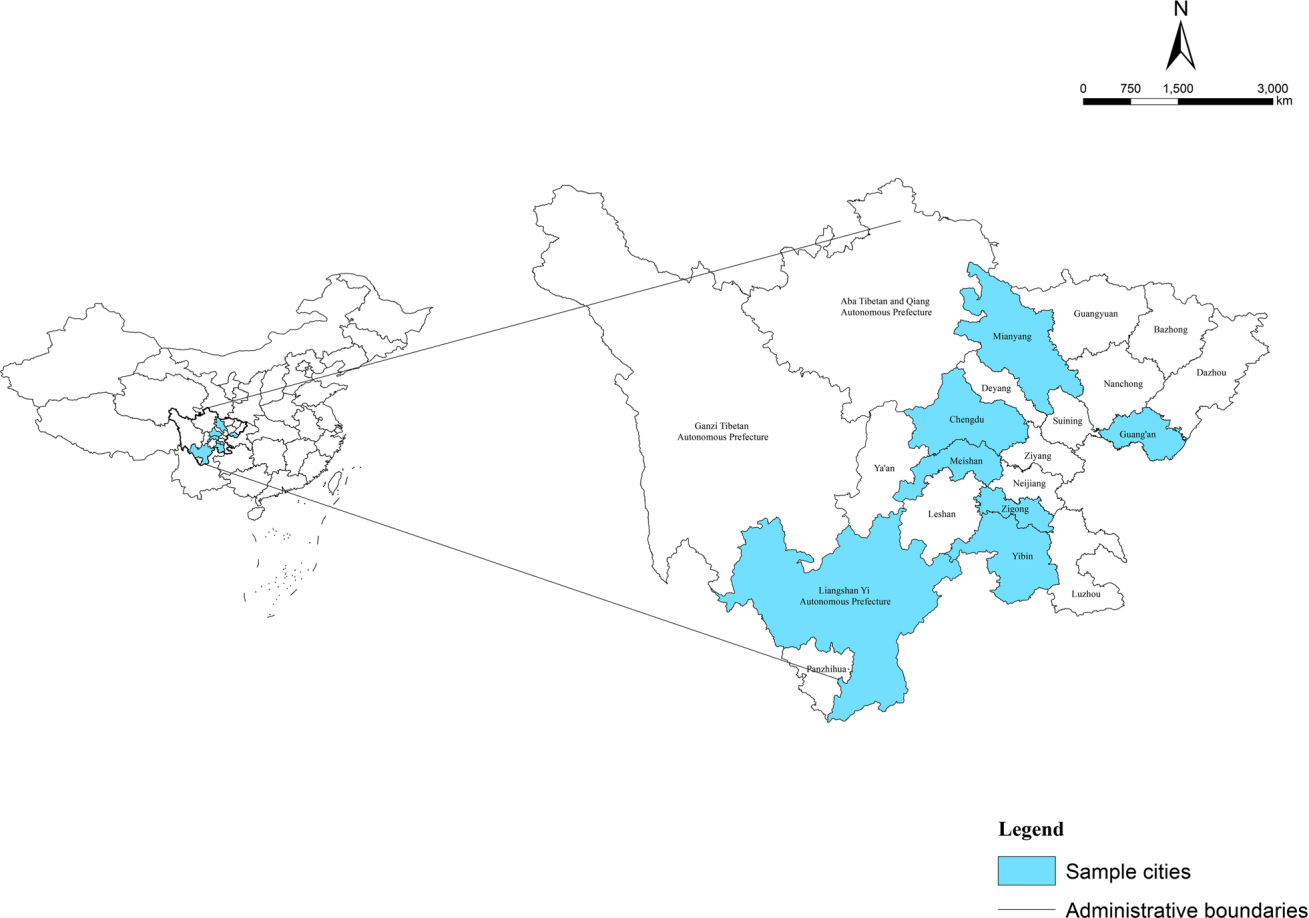
Geographical distribution of 7 sample cities and prefecture in Sichuan Province

The medical institutions were selected in this study by multistage stratified cluster random sampling, which first identified the monitoring areas and then the monitoring institutions. In the first stage, after a comprehensive analysis of the economic development level, health service status, population size, and geographical location of Sichuan Province, seven cities (autonomous prefecture) were selected—Chengdu (the capital of Sichuan province), Mianyang, Meishan, Guang'an, Zigong, Yibin, and Liangshan (Yi Autonomous Prefecture). In the second stage, one district and three counties (county-level cities) were randomly selected from the above-mentioned cities (autonomous prefecture). Next, from the selected districts and counties (county-level cities), 4–10 streets (townships) were randomly selected, totalling 214 townships and communities, and from each street (township), 2–6 communities (villages) were randomly selected as sample units, totalling 945 communities and villages. See Fig. 1.

After selecting the survey districts and counties, sampling was carried out according to the level and type of medical institutions: general hospitals and traditional Chinese medicine hospitals were half of the same-type institutions in the district and county; specialist hospitals were taken according to type—one of each type; maternal and child health hospital, usually 1 per district and county, were directly included; community or township health service centres, about 8 per district and county were included; community health stations and clinics—each district and county has about 50; in all, a total of 163 hospitals (general, Chinese medicine, and specialist hospitals), 35 maternal and child health hospitals, and 1358 primary health care institutions (community health centres, township health centres, community health stations, and clinics) were included. Details are provided in Additional file 1: Table S1.

### Standardised dataset and quality control

Essential information about patients was sourced from the HIS, which was requested to report individual outpatient and inpatient records. The system registers patient information (patient id, age, date of birth, gender, address), disease information (date of visit, department visited, length of stay, disease name, ICD-10 code), medical cost information (total fees, treatment fees, drug fees, check fees, diagnostic fees, surgery fees, material fees, comprehensive medical service fees and other fees), type of payment ( such as Urban Employee Health Insurance, medical insurance for urban and rural residents, household out-of-pocket) and information on medical institutions (name of the medical institution, type of medical institution, level of the medical institution, and the region to which the medical institution belongs).

We used the 2018 edition of *The Compendium of China's First List of Rare Diseases*, which includes 121 rare diseases and their ICD-10 codes. Based on the ICD-10 codes of 121 rare diseases, we extracted 83 rare diseases from 1556 sample institutions. After excluding incomplete and incorrect information, we obtained 13,981 rare disease patient consultation records, including 10,520 outpatient and 3461 inpatient records. After calculating the CCE of 83 rare diseases using the SHA2011 framework, we selected the 10 rare diseases with the highest CCE as our study sample with the aim of getting health policymakers to prioritize the implementation of appropriate policies. Finally, a total of 9714 valid records (including 7188 outpatient records and 2526 inpatient records) for patients with rare diseases were collected, which formed the sample dataset of this study.

These 10 rare diseases are Hemophilia-D66.01, Young-onset Parkinson’s disease (YOPD)-G20, Systemic Sclerosis (SSc)-M34.0, Neuromyelitis Optica (NMO)-G36.0, Autoimmune Encephalitis (AE)-G04.8, Idiopathic Pulmonary Fibrosis (IPF)-J84.1, Multiple System Atrophy (MSA)-G90.3, Multiple Sclerosis (MS)-G35.0, Amyotrophic Lateral Sclerosis (ALS)-G12.2, and Congenital Scoliosis (CS)-Q76.3. When we identified Young-onset Parkinson's disease patients, in addition to selecting ICD-10 codes as G20, we also restricted their age to under 50 years. All analyses and calculations were performed with STATA14.0 (Stata Corporation, College Station, TX, USA).

### Measurement methodology

We have adopted top-down accounting principles to estimate the CCE of rare diseases. Based on the total macro data for Sichuan Province, we used the sample dataset to obtain allocation parameters by which to apportion the total CCE of Sichuan Province in different dimensions. The scope of the CCE measurement includes curative income (CI), government basic expenditure subsidies (BS), and government special project expenditure subsidies (PS).

In the SHA2011 framework, curative care includes outpatient care, inpatient care, rehabilitative care, and long-term health care, but not preventive care and capital formation costs (investments in infrastructure and equipment) in health costs. In China, the data on rehabilitative and long-term care are often mixed with outpatient or inpatient care; it is difficult to separate them. Therefore, rehabilitation care income and long-term care income were not measured separately and were combined with outpatient and inpatient service income, collectively called curative income [22]. BS represents government subsidies for medical services provided by medical institutions, while PS refers to government subsidies for special purposes, such as maternal and childbirth subsidies, basic drug subsidies, or assistance for psychiatric patients.

The calculation process was divided into three steps, which is exemplified in the case of the outpatient medical service. 

*Step 1*. Was to determine the total CCE of medical institutions in Sichuan Province.

1 $$ {\text{CCE}} = {\text{ CI}} + {\text{BS}} + {\text{PS}} $$

where CI and BS were obtained with the following formulas:

2 $$ CI = TOI \times \left( {1 - POI/OI} \right) $$

3 $$ {\text{BS}} = {\text{BCS}} \times \left( {1 - \frac{IBD}{{IBD + COV \times K}}} \right) $$

4 $$ {\text{COV}} = {\text{TOV}} \times \left( {1 - POV/OV} \right) $$

Unlike inpatient care, outpatient care provides both curative service and preventive service. For services of a preventive nature, such as family planning, maternal healthcare, child healthcare, immunization programs, and chronic disease management falling within the scope of preventive services, the charges are recorded in the outpatient income and need to be excluded. In formula (2): TOI denotes the total outpatient income in Sichuan Province in the year 2018; POI refers to the preventive outpatient income of the sample medical institutions, and was calculated by summing the income of all samples that contained preventive services based on ICD-10 codes (Z00, Z01, Z02, etc.). The total outpatient income of sample medical institutions is represented by OI.

In formula (3), BCS denotes the total government basic expenditure subsidies in Sichuan Province in 2018; IBD represents the total number of inpatient bed days, COV refers to the total number of curative outpatient visits, and K takes a constant value of 0.1 following the recommendations of the China National Health Development Research Center, meaning that 10 outpatient visits are equivalent to 1 inpatient bed day [23]. COV was calculated using formula (4), where TOV denotes the total number of outpatient visits in Sichuan Province in 2018, and POV and OV represent preventive services and total outpatient visits in the sample data, respectively.

*Step 2*. Calculation of the CCE per patient.

In line with the “top-down” approach, the CCE per patient among medical institutions in Sichuan was calculated with the following formula:

5 $$ S_{CI} = CI \times \frac{EOI}{{\mathop \sum \nolimits_{i = 1}^{n} EOI_{i} }}\quad \left( {{\text{i}} = 1,2,3 \ldots {\text{n}}} \right) $$

6 $$ S_{BS} = BS \times \frac{EOV}{{\mathop \sum \nolimits_{m = 1}^{n} EOV_{m} }}\quad \left( {{\text{m}} = 1,2,3 \ldots {\text{n}}} \right) $$

7 $$ S_{PS} = PS \times \frac{EPS}{{\mathop \sum \nolimits_{w = 1}^{n} EPS_{w} }}\quad \left( {{\text{w}} = 1,2,3 \ldots {\text{n}}} \right) $$

In formulas (5), (6), and (7), SCI, SBS, and SPS represent curative expenditure, government basic expenditure subsidy, and government special project expenditure subsidy per patient in Sichuan Province. EOI and EPS represent outpatient expenditure and project expenditure per patient in the sample institutions, and EOV represents the number of visits per patient.

*Step 3*. Summary of the CCE with different characteristics.

Formula (8) is the summary of CCE of patients with the same characteristics, such as age, gender, and disease in the region.

8 $$ \sum\nolimits_{i = 1}^{n} {S_{CCE} } = \sum\nolimits_{i = 1}^{n} {S_{CI} } + \sum\nolimits_{i = 1}^{n} {S_{BS} } + \sum\nolimits_{i = 1}^{n} {S_{PS} } \left( {i = 1,2,3 \ldots n} \right) $$

Last, different dimensions of the rare disease CCE in Sichuan Province in 2018 were obtained based on classification and summary of financing source, health service providers, and health spending by beneficiary.

## Results

### General statistics of patients with the 10 rare diseases

In 2018, there were 9714 cases of the 10 rare diseases in Sichuan Province, including 7188 outpatients and 2526 inpatients. Of them, SSc patients totaled 2534 (26.09%) and patients with YOPD and Hemophilia totaled 1935 (19.92%) and 1504 (15.48%), respectively. The average age of patients was 42.80, of which the average age of patients with CS was the lowest (13.28 years) and the highest was for IPF patients (67.91 years). In terms of gender distribution, there were more women (55.14%) than men (44.86%). Five diseases, such as SSc and NMO, mainly afflicted females, while male patients were usually diagnosed with the other five diseases, such as Hemophilia and IPF (Table 1).

**Table 1:** The general statistics of patients with 10 rare diseases in Sichuan Province in 2018 (n/%)

Disease	Total number of patients	Average age ($\overline{x }\pm$ s)	Gender		Service function	
			Male	Female	Outpatients	Inpatients
SSc	2534 (26.09)	49.02 ± 15.26	565 (22.30)	1969 (77.70)	2180 (86.03)	354 (13.97)
YOPD	1935 (19.92)	42.83 ± 9.50	1030 (53.23)	905 (46.77)	1744 (90.13)	191 (9.87)
Hemophilia	1504 (15.48)	18.10 ± 17.03	1437 (95.55)	67 (4.45)	954 (63.43)	550 (36.57)
NMO	1340 (13.79)	46.32 ± 14.40	178 (13.28)	1162 (86.72)	1017 (75.90)	323 (24.10)
MS	682 (7.02)	41.35 ± 13.92	195 (28.59)	487 (71.41)	416 (61.00)	266 (39.00)
MSA	571 (5.88)	62.77 ± 9.34	311 (54.47)	260 (45.53)	397 (69.53)	174 (30.47)
ALS	415 (4.27)	57.27 ± 10.99	241 (58.07)	174 (41.93)	172 (41.45)	243 (58.55)
AE	323 (3.33)	33.47 ± 18.49	158 (48.92)	165 (51.08)	131 (40.56)	192 (59.44)
IPF	280 (2.88)	67.91 ± 10.31	186 (66.43)	94 (33.57)	103 (36.79)	177 (63.21)
CS	130 (1.34)	13.28 ± 10.28	57 (43.85)	73 (56.15)	74 (56.92)	56 (43.08)
Total	9714 (100.00)	42.80 ± 19.06	4358 (44.86)	5356 (55.14)	7188 (74.00)	2526 (26.00)

*AE* autoimmune encephalitis, *ALS* amyotrophic lateral sclerosis, *CS* congenital scoliosis, *IPF* idiopathic pulmonary fibrosis, *MS* multiple sclerosis, *MSA* multiple system atrophy, *NMO* neuromyelitis optica, *SSc* systemic sclerosis, *YOPD* young-onset Parkinson’s disease

### CCE and average expenditure per visit of the 10 rare diseases

The CCE of the 10 rare diseases totalled $19.001 million, with outpatient CCE of $5,686,600 (29.93%) and inpatient CCE of $13,314,500 (70.07%). The three diseases with the highest CCE were Hemophilia ($4,378,600), YOPD ($2,964,700), and SSc ($2,445,700). The average outpatient expenditure per visit for the 10 rare diseases was $109.33 and the top three rare diseases were AE ($308.36), Hemophilia ($221.54), and NMO ($120.04). The average outpatient expenditure per visit for rare diseases, except CS, was spent mainly on drugs. The average inpatient CCE per visit was $2411.64, of which CS expenditure was the highest ($10,491.82), which was 3.08 times the per capita disposable income of Sichuan residents. (Table 2).

**Table 2:** The CCE, average expenditure and drug cost per visit for 10 rare diseases in Sichuan Province in 2018

Disease	Outpatients			Inpatients			Total
	CCE $Million (%)	Average expenditure per visit ($)	Average drug cost per visit (%)	CCE $Millio (%)	Average expenditure per visit ($)	Average drug cost per visit (%)	CCE $Million (%)
Hemophilia	154.73 (35.34)	221.54	86.76	283.13 (64.66)	2112.22	45.35	437.86 (23.04)
YOPD	102.07 (34.35)	74.50	87.12	194.2 (65.55)	5141.82	6.57	296.27 (15.59)
SSc	144.35 (59.02)	88.78	70.03	100.22 (40.98)	1180.98	35.54	244.57 (12.87)
NMO	80.65 (34.92)	120.04	89.11	150.29 (65.08)	2104.93	30.65	230.94 (12.15)
AE	25.63 (14.47)	308.36	51.78	151.49 (85.53)	3766.57	33.14	177.12 (9.32)
IPF	6.67 (5.42)	82.97	77.38	116.28 (94.58)	2781.55	33.86	122.95 (6.47)
MS	16.75 (15.82)	52.02	64.76	89.15 (84.18)	1481.66	31.32	105.91 (5.57)
CS	4.06 (4.14)	82.96	0.01	93.99 (95.86)	10,491.82	5.15	98.05 (5.16)
MSA	27.86 (28.45)	110.31	86.82	70.06 (71.55)	1515.47	19.78	97.93 (5.15)
ALS	5.89 (6.65)	49.30	75.91	82.64 (93.35)	1601.52	16.21	88.53 (4.66)
Total	568.66 (29.93)	109.33	79.50	1331.45 (70.07)	2411.64	26.65	1900.11 (100.00)

*CCE* curative care expenditure

### Health financing schemes for the ten rare diseases

**Fig.2:**
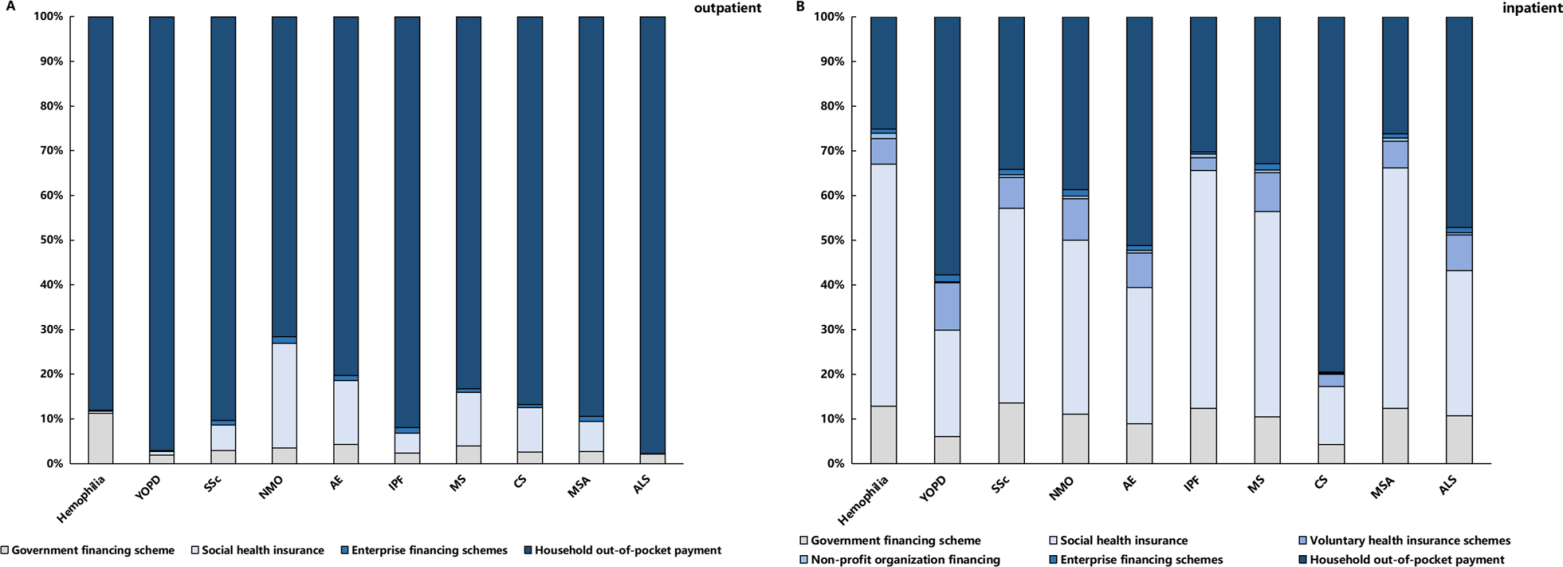
Outpatient (**A**) and inpatient (**B**) CCE financing schemes for the 10 rare diseases in Sichuan Province in 2018 (%)

As shown in Fig. 2, 86% of outpatient CCE was financed from household out-of-pocket (OOP) expenditure, while social health insurance, the secondary source, only accounted for 7.85% of CCE. In outpatient CCE, the OOP of CS was the highest (97.73%) while the OOP of YOPD was the lowest (71.64%). The household OOP expenditure, the primary source of financing, accounted for 41.60% of inpatient expenditure, while social health insurance (39.58%) was the secondary financing source. Unlike outpatients, inpatients had access to six financing sources, which are relatively more diversified. In terms of inpatient services, the OOP for CS was the highest (79.6%), while OOP for YOPD and AE were also more than 50%, all of which imposed a heavy economic burden on patients.

### Allocation of CCE in different medical institutions for the 10 rare diseases

**Fig. 3:**
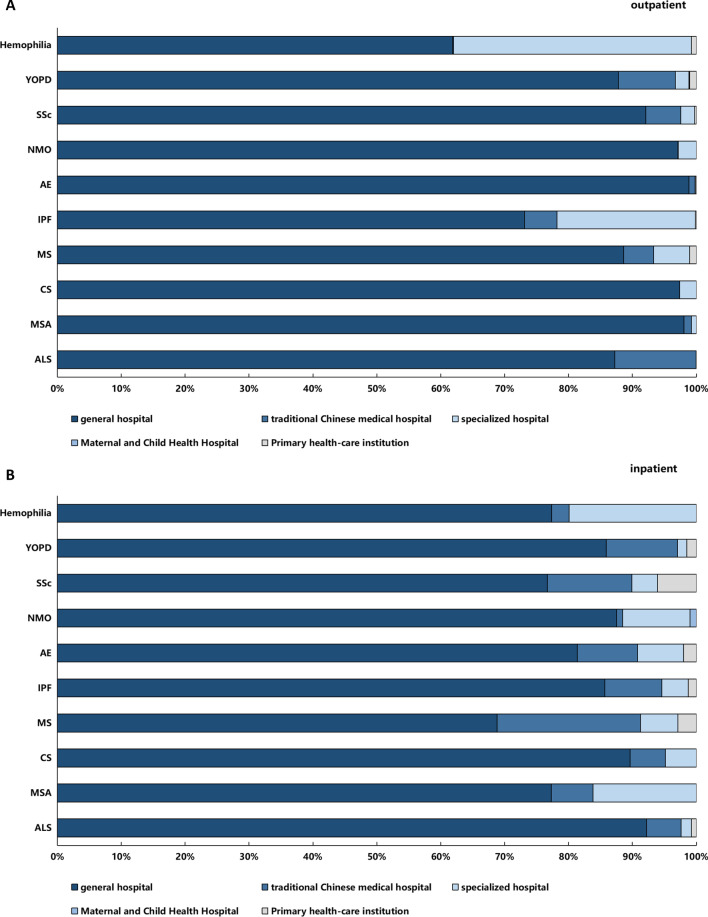
Allocation of outpatient (**A**) and inpatient (**B**) CCE in different medical institutions in Sichuan Province in 2018 (%)

Figure 3 shows that more than 80% of the rare disease CCE was incurred in general hospitals, followed by specialized hospitals (outpatient 11.70%, inpatient 8.81%), and Chinese traditional medicine hospitals (outpatient 3.55%, inpatient 7.74%). The outpatient CCE of three diseases (Hemophilia, YOPD, and MS) and the inpatient CCE of two diseases (CCE of SSc and NMO) were incurred in primary medical institutions, indicating that such institutions also provided some rare disease treatment services.

### The CCE of the 10 rare diseases by beneficiary

**Fig. 4:**
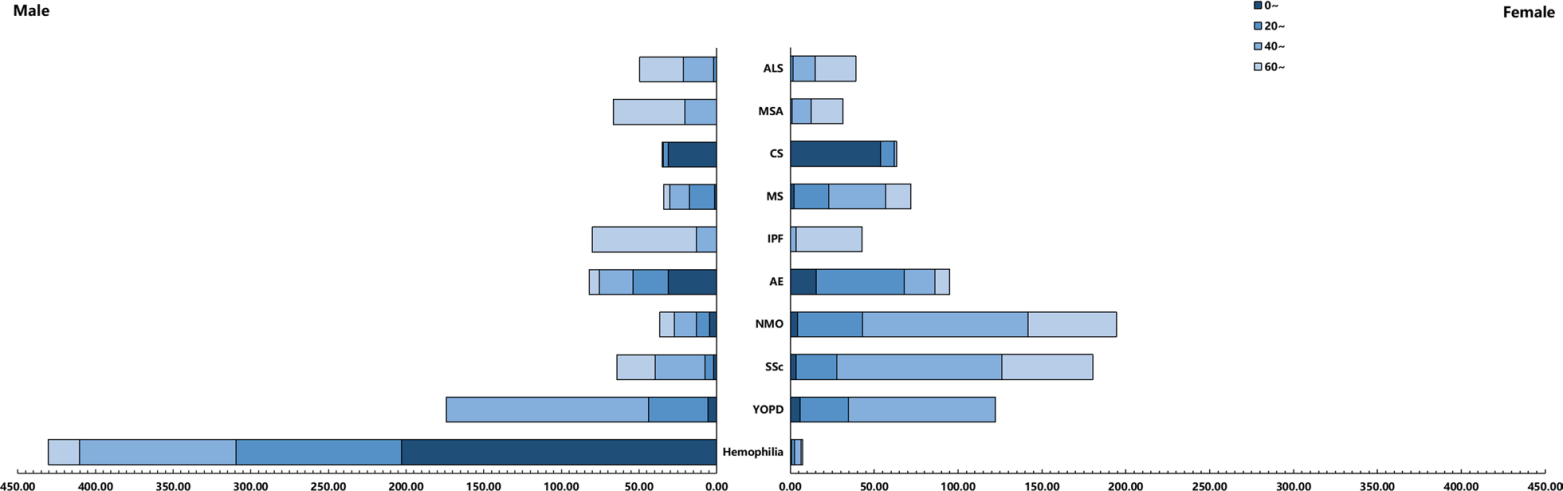
The CCE of the 10 rare diseases by beneficiary in Sichuan Province in 2018 ($ Million)

The CCE of Hemophilia and CS was mainly in children and adolescents; CCE of YOPD, SSc, NMO, and MS were mainly among people aged 40 ~ years, and the CCE of IPF and MSA were mainly distributed among people aged 60 ~ years. Almost all the CCE of Hemophilia, IPF, and MSA were on men. (Fig. 4).

## Discussion

In this study, the CCE of the 10 rare diseases in Sichuan Province in the year 2018 was estimated based on the SHA2011 accounting framework. The results show that the CCE of the 10 rare diseases total $19.001 million, accounting for 0.06% of the CCE in Sichuan Province in 2018 and the average outpatient expenditure per visit was $109.33, accounting for 3.22% of the per capita disposable income in Sichuan Province in 2018 and 1.40% of per capita GDP. The average inpatient expenditure per visit was $2411.64, accounting for 70.99% of per capita disposable income and 30.93% of GDP per capita. These results cannot be compared with the findings of studies done in other countries because only a few were based on the SHA2011 accounting framework or used such a top-down approach to calculate rare disease CCE. Further, the definition of rare diseases differs from country to country. Nevertheless, our study provides a new approach for calculating the CCE of rare diseases and can be used for formulating policy measures.

Except for MS and ALS, the average outpatient expenditure per visit of all other rare diseases in our study were higher than average outpatient expenditure per visit for diabetes in China by $70.65 [24]; The average expenditure per visit for nine rare diseases, including Hemophilia, were much higher than the average expenditure per visit for diabetes in China in the same year ($1210) [25]. Furthermore, the average expenditure per visit for YOPD, AE, and CS were higher than the average expenditure per visit for cancer, such as gastric, oesophageal, breast, and lung cancer [26, 27]. The high average expenditure per visit and OOP, especially outpatient expenditure, indicate that rare diseases impose a high economic burden on patients and their families, which can easily push them into poverty. In China, the high R&D costs and low returns on rare disease drugs (orphan drugs) make pharmaceutical companies reluctant to invest in these drugs. Most rare disease drugs on the Chinese market are imported and therefore, are expensive, posing a considerable economic challenge for patients [28–30]. Effective measures must be adopted to ensure that rare disease patients can access affordable drugs. In recent years, the Chinese government has initiated many policy measures to improve the accessibility of health services for rare disease patients [31–33]. In 2017, the National Healthcare Security Administration of China included recombinant human coagulation factor VIIa for injection in the treatment of Hemophilia, recombinant human interferon β-Ib in the treatment of MS, and pirfenidone for the treatment of IPF in the Medicine List for National Basic Medical Insurance and Employment Injury Insurance and Maternity Insurance (or The National Reimbursement Drug List) [34]. The inpatient OOP for these three rare diseases was found to be low in our study, indicating that the policies may have been effective. However, the outpatient OOP for rare diseases remains high and the amount of reimbursement in medical insurance is very low. Studies have found that many restrictions continue on medical insurance coverage for outpatients [35, 36]. Further, the medical insurance reimbursement policy in China focuses more on rare disease drug cost-sharing, while non-drug treatment-based rare diseases cost more but are noticed less by policymakers. This explains the high OOP for CS patients, who depend mainly on surgery for treatment. Therefore, policymakers should look at reducing the disease burden of rare disease patients who rely on non-drug treatments.

We suggest exploring newer and more diversified medical security systems for rare diseases that are co-paid by different parties to alleviate the economic burden on rare disease patients. The social health insurance system should play a basic role; more rare disease treatment drugs should be incorporated into the National Reimbursement Drug List. Additionally, a multi-tiered healthcare security system covering critical illness insurance, medical assistance, commercial insurance, and social assistance should be established so that rare disease patients can access multiple channels of cost-sharing [37]. Most importantly, when formulating health insurance payment standards and multi-party co-payment mechanisms, we should consider the restrictions on rare disease reimbursement. For example, it is necessary to set an appropriate threshold, an annual limit of health insurance payment, and the top limit of annual OOP CCE based on actual CCE levels for different rare diseases in different regions to ensure that patients can benefit from the policies.

As with most diseases, rare disease treatment services are mainly provided by general hospitals because of their competence in diagnosis and treatment. China's primary medical institutions are limited in their technology level and, therefore, focus on the diagnosis and treatment of common and frequently-occurring diseases. However, our study found that primary medical institutions also undertake the treatment of some rare diseases. For example, they provide outpatient services for SSc and MS patients as well as inpatient services for YOPD patients. This shows that it is feasible to improve doctors' expertise in the diagnosis and treatment of rare diseases in primary medical institutions. We recommend providing more training to the doctors to identify rare diseases and enabling them to screen, refer, and treat rare diseases, thus effectively shortening the diagnostic cycle of rare diseases and reducing health care resource consumption and disease-induced economic burden [38, 39].

The CCE distribution trend of rare diseases in the population is approximately the same as the prevalence trend seen in this study [40–45]. For example, the prevalence rate of Hemophilia is relatively high, and it mainly affects boys. YOPD, IPF, and MSA are more common in middle-aged and elderly people, while NMO and MS are more common in women. Consequently, these groups consume more CCE. As rare diseases are mostly genetic, effective prevention is more important than treatment [46]. We should focus on women of childbearing age and new-borns with a family history of rare disease. Tertiary prevention before, during, and after pregnancy are essential for reducing rare diseases. First, it is necessary to perform genetic screening before pregnancy and make appropriate reproductive choices based on genetic counselling, pathogenic gene carrier screening, and risk assessment. Second, it is important to carry out prenatal screening and diagnosis to avoid giving birth to at-risk babies as much as possible. For newborns with a family history of rare disease, genetic screening, early diagnosis and treatment, and increased lifetime follow-up management and drug control are important in improving the patient's quality of life. IPF is the only respiratory disease among the ten rare diseases that we evaluated. Raghu [47] found that IPF is more common in elderly men with a history of smoking. Therefore, educating people about adopting and maintaining healthy lifestyles is also essential for the prevention and control of rare diseases [48, 49].

This study has the following limitations. The results we measured may only be the tip of the iceberg. First, the data from the HIS did not consider the out-of-hospital purchase of medication and other expenses of patients. Further, due to the hereditary, chronic, and progressive nature of most rare diseases, the vast majority of patients require repeated hospitalization; if the number of hospitalizations is included at the current costs, the economic burden on patients would be much higher. Second, we may have underestimated the actual scale of rare disease CCE. When selecting hospitalised patients, this study used the first diagnosis as the data; there may be some rare disease patients who were not included in the calculation. Last, we did not include misdiagnosed rare disease patients and those who were not diagnosed or not clearly diagnosed. Despite these limitations, this study is a rare comprehensive report covering the financing scheme of rare disease CCE, health provider distribution, and beneficiary groups. It can help improve the understanding of the impact of rare diseases on Chinese society by providing empirical evidence for rare disease research.

## Conclusions

The high expenditure on rare diseases has always been a controversial topic, posing a considerable challenge to patients, health policymakers, health care providers, and society. So far, there is neither an international standard to assess the scale of rare disease CCE nor research on the financing structure of rare disease CCE. Using the SHA2011 framework, this study calculated the CCE of the 10 rare diseases in Sichuan Province on macro and micro levels and provided details on the total amount of CCE, financing schemes, health providers, and beneficiary groups. Our results show that the total CCE of the 10 rare diseases is $19,001,100, the average CCE per visit is high and the patients’ have to bear a heavy economic burden. CCE are mainly incurred in general hospitals and distributed among the 40–59 age group, indicating that the financing structure of rare disease CCE must be optimised and that a rare disease prevention and treatment model based on multi-party collaboration and financing should be explored to improve the level of medical security for rare disease patients. In addition, it is essential to enhance the ability of primary medical institutions to diagnose some rare diseases and build a three-tiered rare disease prevention system.

## Supplementary Information

**Additional file 1.** Distribution of sample institutions in Sichuan Province in 2018.

## Data Availability

The datasets generated during and/or analyzed during the current study are available from the corresponding author on reasonable request.

## Abbreviations

CCE: Curative care expenditure
OOP: Out-of-pocket
SHA2011: System of health accounts 2011
YOPD: Young-onset Parkinson’s disease
SSc: Systemic sclerosis
NMO: Neuromyelitis optica
AE: Autoimmune encephalitis
IPF: Idiopathic pulmonary fibrosis
MSA: Multiple system atrophy
MS: Multiple sclerosis
ALS: Amyotrophic lateral sclerosis
CS: Congenital scoliosis
HIS: Hospital information system
