# Supplementary material for: Rare disease curative care expenditure-financing scheme-health provider–beneficiary group analysis: an empirical study in Sichuan Province, China
Source: Orphanet J Rare Dis. 2022 Oct 8;17:373. doi: 10.1186/s13023-022-02524-1 (PMC9548194; doi:10.1186/s13023-022-02524-1)
Supplement: Supplementary file 1 — Additional file 1. Distribution of sample institutions in Sichuan Province in 2018. [file 13023_2022_2524_MOESM1_ESM.docx]

**Table 1** Distribution of Sample institutions in Sichuan Province in 2018

| Sample Region | | Hospitals | Community Health Centres and Township Health Centres | Community Health Stations and Clinics | Maternal And Child Health Hospitals | Total |
| --- | --- | --- | --- | --- | --- | --- |
| Provincial medical institution | | 16 | 0 | 0 | 0 | 16 |
| Chengdu | Municipal level | 9 | 0 | 0 | 1 | 10 |
|  | Jintang County | 6 | 8 | 46 | 1 | 61 |
|  | Longquanyi District | 6 | 9 | 41 | 1 | 57 |
|  | Shuangliu District | 6 | 8 | 34 | 1 | 49 |
|  | Xinjin County | 5 | 8 | 35 | 1 | 49 |
| Guang'an | Municipal level | 3 | 0 | 0 | 1 | 4 |
|  | Guang'an District | 4 | 8 | 44 | 1 | 57 |
|  | Huaying City | 2 | 8 | 40 | 1 | 51 |
|  | Wusheng County | 4 | 7 | 48 | 1 | 60 |
|  | Yuechi County | 4 | 8 | 51 | 1 | 64 |
| Meishan | Municipal level | 4 | 0 | 0 | 1 | 5 |
|  | Dongpo District | 1 | 8 | 50 | 1 | 60 |
|  | Pengshan District | 5 | 8 | 50 | 1 | 64 |
|  | Qingshen County | 4 | 8 | 43 | 1 | 56 |
|  | Renshou County | 4 | 9 | 52 | 1 | 66 |
| Mianyang | Municipal level | 4 | 0 | 0 | 1 | 5 |
|  | Fucheng District | 7 | 8 | 47 | 1 | 63 |
|  | Jiangyou City | 8 | 8 | 43 | 1 | 60 |
|  | Santai County | 5 | 10 | 50 | 1 | 66 |
|  | Youxian District | 3 | 8 | 48 | 1 | 60 |
| Yibin | Municipal level | 4 | 0 | 0 | 1 | 5 |
|  | Cuiping District | 3 | 8 | 39 | 1 | 51 |
|  | Jiang'an County | 3 | 8 | 50 | 1 | 62 |
|  | Pingshan County | 3 | 8 | 50 | 1 | 62 |
|  | Changning County | 3 | 8 | 50 | 1 | 62 |
| Zigong | Municipal level | 7 | 0 | 0 | 1 | 8 |
|  | Fushun County | 8 | 8 | 46 | 1 | 63 |
|  | Gongjing District | 2 | 8 | 51 | 1 | 62 |
|  | Rong County | 5 | 7 | 35 | 1 | 48 |
|  | Yantan District | 2 | 8 | 51 | 1 | 62 |
| Liangshan | Municipal level | 3 | 0 | 0 | 1 | 4 |
|  | Dechang County | 2 | 6 | 10 | 1 | 19 |
|  | Ningnan County | 3 | 4 | 17 | 1 | 25 |
|  | Xichang City | 3 | 5 | 12 | 1 | 21 |
|  | Xide County | 2 | 5 | 11 | 1 | 19 |
| Total | | 163 | 214 | 1144 | 35 | 1556 |
